# Host, Pathogen, and Environmental Characteristics Predict White-Nose Syndrome Mortality in Captive Little Brown Myotis (*Myotis lucifugus*)

**DOI:** 10.1371/journal.pone.0112502

**Published:** 2014-11-19

**Authors:** Joseph S. Johnson, DeeAnn M. Reeder, James W. McMichael, Melissa B. Meierhofer, Daniel W. F. Stern, Shayne S. Lumadue, Lauren E. Sigler, Harrison D. Winters, Megan E. Vodzak, Allen Kurta, Joseph A. Kath, Kenneth A. Field

**Affiliations:** 1 Department of Biology, Bucknell University, Lewisburg, Pennsylvania, United States of America; 2 Department of Biology, Eastern Michigan University, Ypsilanti, Michigan, United States of America; 3 Illinois Department of Natural Resources, Springfield, Illinois, United States of America; CSIRO, Australia

## Abstract

An estimated 5.7 million or more bats died in North America between 2006 and 2012 due to infection with the fungus *Pseudogymnoascus destructans* (*Pd*) that causes white-nose syndrome (WNS) during hibernation. The behavioral and physiological changes associated with hibernation leave bats vulnerable to WNS, but the persistence of bats within the contaminated regions of North America suggests that survival might vary predictably among individuals or in relation to environmental conditions. To investigate variables influencing WNS mortality, we conducted a captive study of 147 little brown myotis (*Myotis lucifugus*) inoculated with 0, 500, 5 000, 50 000, or 500 000 *Pd* conidia and hibernated for five months at either 4 or 10°C. We found that female bats were significantly more likely to survive hibernation, as were bats hibernated at 4°C, and bats with greater body condition at the start of hibernation. Although all bats inoculated with *Pd* exhibited shorter torpor bouts compared to controls, a characteristic of WNS, only bats inoculated with 500 conidia had significantly lower survival odds compared to controls. These data show that host and environmental characteristics are significant predictors of WNS mortality, and that exposure to up to 500 conidia is sufficient to cause a fatal infection. These results also illustrate a need to quantify dynamics of *Pd* exposure in free-ranging bats, as dynamics of WNS produced in captive studies inoculating bats with several hundred thousand conidia may differ from those in the wild.

## Introduction

White-nose syndrome (WNS) is a fungal disease affecting hibernating bats, causing the death of an estimated 5.7–6.7 million bats since its initial discovery in North America in 2006 (USFWS 2012). WNS is caused by the cold-adapted fungus *Pseudogymnoascus destructans* (*Pd*), which invades the dermis and epidermis of bats during hibernation [1]–[3]. Bats are vulnerable to *Pd* because their immune system is suppressed along with nearly all physiological processes during hibernation [4]–[6], and because the cold temperature and high humidity typical in many bat hibernacula represent ideal conditions for *Pd* growth [7]–[9]. *Pd* was first identified as *Geomyces destructans* in 2009 [10], and was reclassified to the genus *Pseudogymnoascus* in 2013 [11]. The fungus is not native to North America, and is believed to have been introduced from Europe [12], [13]. Although large-scale mortality of bats has never been documented in Europe, 90% mortality of bats occurs in North American hibernacula after *Pd* is introduced [14], [15]. Such high mortality rates have led to predictions of regional extinctions, although not all bat species appear equally affected by the disease [15], [16].

The little brown myotis (*Myotis lucifugus*) is among the species most heavily impacted by WNS [15]. Little brown myotis infected with *Pd* arouse more frequently from hibernation than unaffected bats, resulting in exhaustion of fat reserves needed to survive the winter [13], [17]. Although the trigger for this increase in arousals has yet to be confirmed, there is evidence that hypotonic dehydration of infected bats may influence arousal behaviors [18]. Thus, it is likely that little brown myotis are particularly vulnerable to *Pd* because they have naturally high rates of evaporative water loss during winter, and because their small size (<10 g) limits their total fat reserves upon entering hibernation and the number of periodic arousals that can be sustained during winter [19]–[21].

Despite the high mortality rates of little brown myotis inhabiting *Pd*-contaminated hibernacula, summer maternity colonies of little brown myotis still persist within the contaminated region of North America. The persistence of these maternity colonies, which are typically composed of female bats that return to familiar roosts each year after hibernation [22]–[24], suggests that some bats have survived several winters of exposure to *Pd*. Although it is possible that these bats over-winter in hibernacula still unexposed to *Pd* or survived due to random factors, it is more likely that some bats have naturally higher survival rates when exposed to the fungus. This survival may result from immunological resistance to *Pd*, differences in physiology (e.g., larger body size or lower rate of evaporative water loss), or behavioral ecology (e.g., use of hibernaculum microclimates less favorable for fungal growth) that result in higher resilience. Studies of the little brown myotis demonstrate individual variability in both winter ecology and physiology, including differences in torpor patterns and energy use, and selection of microclimates within a hibernaculum, representing possible foundations for variation in survival [25]–[27].

If individual variation in ecology and physiology relate to WNS mortality, then mortality should vary predictably. For example, male little brown myotis utilize their winter energy reserves more rapidly than females [26], [27], potentially making them more vulnerable to the further depletion of fat reserves when exposed to *Pd*. Mortality may also vary among bats inhabiting different hibernacula or areas within a hibernaculum that have different microclimates. Higher population declines have been observed among populations of little brown myotis inhabiting warmer hibernacula [28], a trend possibly linked to the growth rate of *Pd*, which peaks between 12.5 and 15.8°C and declines at warmer and colder temperatures [8]. Temperatures inside hibernacula of little brown myotis are typically below 10°C but range widely, including environmental conditions with varying suitability for *Pd* growth [7], [29]. Because temperature also affects the winter torpor behaviors and energy expenditure of bats [30], [31], variables such as the temperature and individual behavior and physiology are likely to have interacting effects on fungal growth and WNS mortality.

Thus, understanding whether or not some bats are better able to survive WNS requires an understanding of the interaction of the environment, host, and pathogen, a concept presented in the disease triangle [9], [32], [33]. However, our current understanding of WNS mortality lacks such context because laboratory studies investigating the disease are conducted under a single environmental condition, typically exposing bats to 500 000 *Pd* conidia and hibernating them at 7°C, without consideration for individual variation in survival [3], [13].

Our purpose was to examine WNS mortality and survival in a captive population of little brown myotis in the context of this disease triangle. We hypothesized that the number of *Pd* conidia bats are initially exposed to affects fungal load at the end of hibernation, duration of torpor bouts, and mortality. We further hypothesized that bats hibernating at warm temperatures have higher *Pd* loads at the end of hibernation, exhibit shorter torpor bouts, and experience greater mortality. Finally, we hypothesized that mortality would be inversely related to body condition at the onset of hibernation, and that mortality would be greatest among males.

## Results

**Figure 1:**
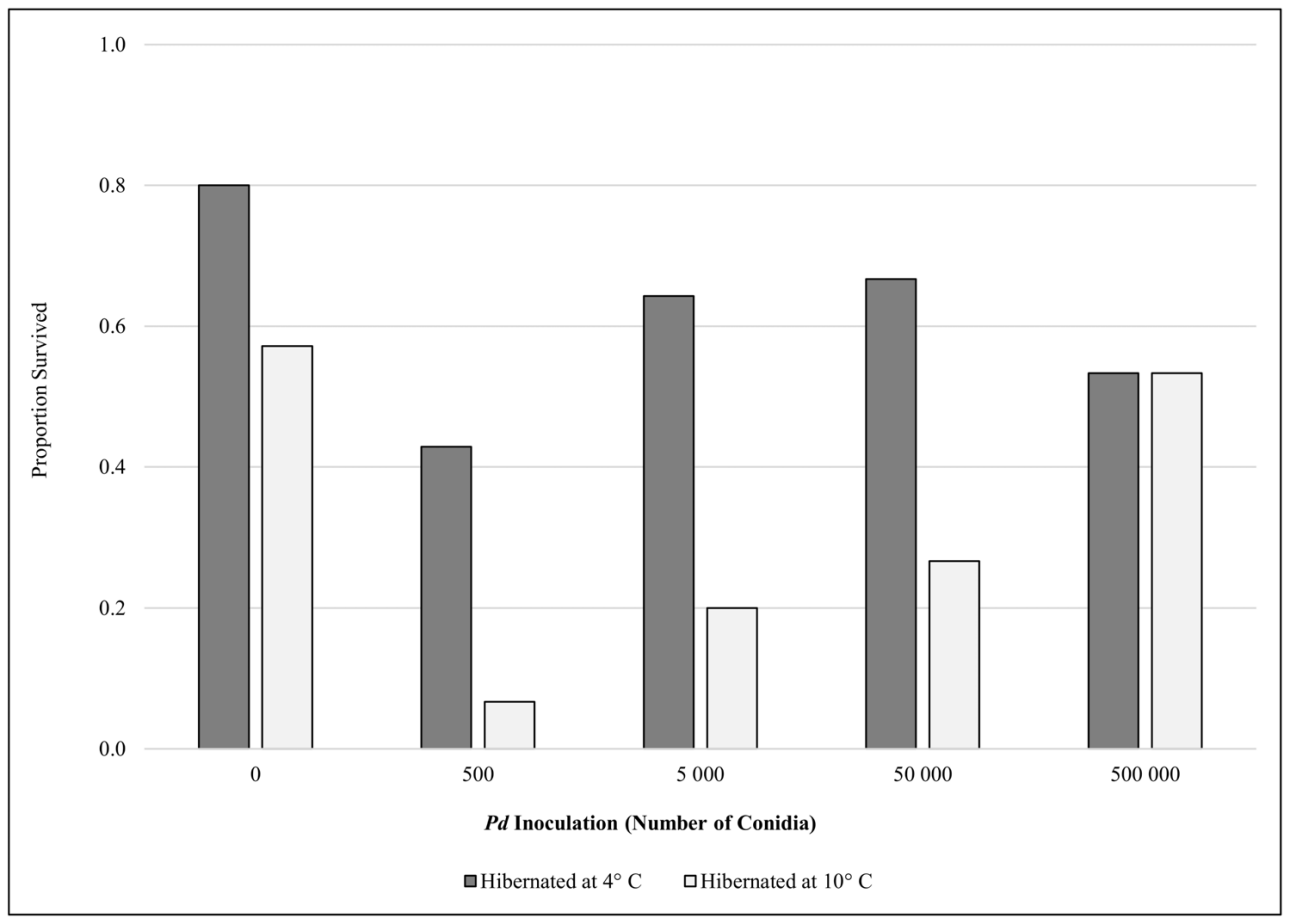
Comparison of survival rates (percent) for little brown myotis (*Myotis lucifugus*) inoculated with different doses of *Pseudogymnoascus destructans* (*Pd*) conidia and hibernated for five months at either 4 or 10°C.

Of the 147 little brown myotis, 69 (47%) survived the five-month captive hibernation study. Three emaciated bats died within 1 week of removal from hibernation and were not considered to have survived hibernation. Logistic regression analysis showed that mortality among little brown myotis was not random (χ^2^  = 65.9, df  = 6, *P*<0.001), with *Pd* inoculation (*P* = 0.037), temperature (*P*<0.001), sex (*P* = 0.024), and pre-hibernation body condition (*P*<0.001) significantly influencing mortality (Fig. 1). Mortality in control groups consisted mostly (89%) of males (3 of 3 bats in the 4°C control; 5 of 6 bats in the 10°C control), all of which had body condition indices at the onset of hibernation that were below the median. Among inoculation treatments, only bats exposed to 500 *Pd* conidia had regression coefficients significantly different from 0, meaning it was the only inoculation treatment with mortality odds significantly greater than the control groups (Table 1). Mortality odds for males were significantly greater than females, greater for bats hibernating at 10°C compared to 4°C, and initial body condition had a negative, linear effect on mortality (Table 1). Average body condition of females (0.224±0.02 s.d.) was significantly higher (*t* = −6.0, df 145, *P*<0.001) than males (0.206±0.02) at the onset of hibernation, but the correlation between body condition and sex in the logistic regression model (0.255) was not great enough to merit removal of either variable. Thus, host and environmental variables helped predict mortality, with males, bats of both sexes with low body condition, and bats hibernating at warmer temperatures less likely to survive.

**Table 1:** Logistic regression analysis of little brown myotis (*Myotis lucifugus*) survival when experimentally inoculated with *Pseudogymnoascus destructans* under varying conditions.

Variable	*W*	P-value	Odds Ratio	95% Confidence Interval
Body Condition	15.6	<0.001	0.57	0.43–0.75
Temperature				
10°C	15.6	<0.001	5.8	2.4–14.0
Sex				
Male	5.1	0.024	2.8	1.1–6.8
*Pd* inoculation				
500 spores	8.4	0.004	9.1	2.0–40.4
5 000 spores	1.5	0.217	2.4	0.61–8.8
50 000 spores	0.2	0.663	1.3	0.36–5.1
500 000 spores	0.3	0.609	1.4	0.38–5.6

For categorical variables, results are given in respect to a reference condition of 10°C, female, and inoculated with no fungal spores.

**Figure 2:**
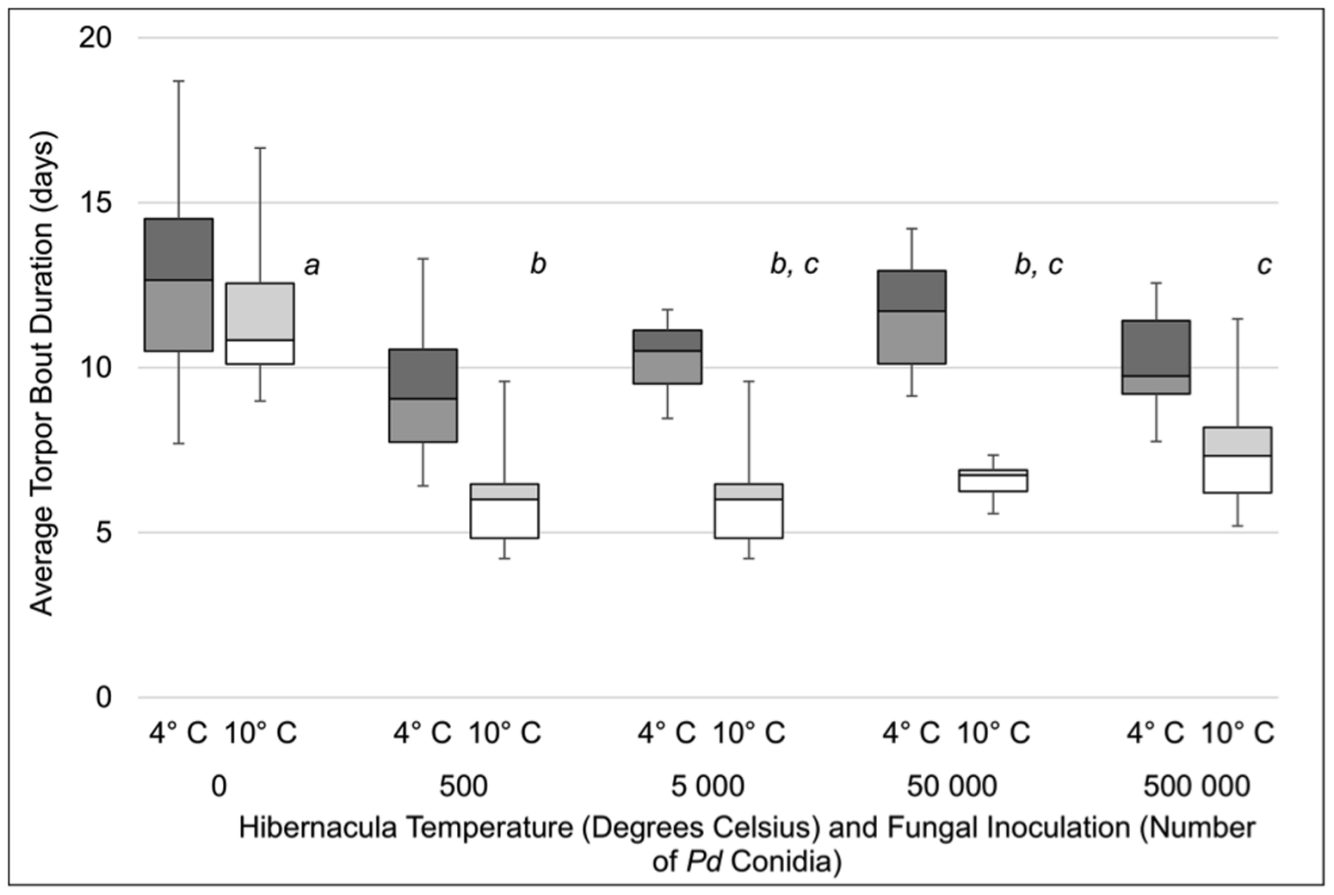
Average duration of torpor bouts (days) for little brown myotis (*Myotis lucifugus*) inoculated with different doses of *Pseudogymnoascus destructans* (*Pd*) conidia and hibernated for five months at either 4 or 10°C. Within each temperature, treatments not sharing common superscript letters were significantly different (*P*<0.05). All doses differed between temperatures (*P*<0.05).

**Figure 3:**
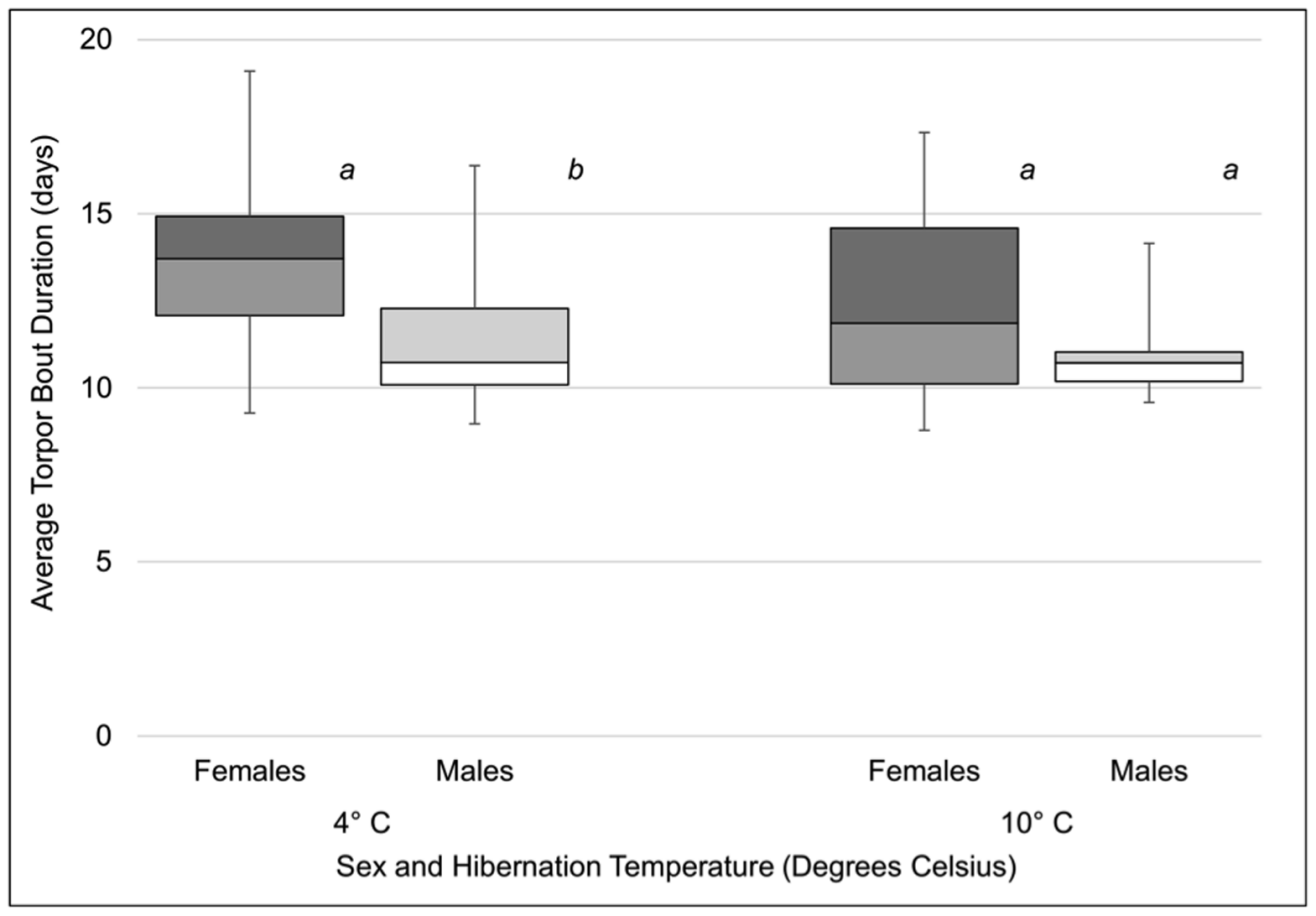
Average duration of torpor bouts (days) of male and female little brown myotis (*Myotis lucifugus*) not inoculated with *Pseudogymnoascus destructans* (*Pd*) and hibernated at either 4 or 10°C. Within each temperature, sexes not sharing common superscript letters were significantly different (*P*<0.05).

Differences in torpor patterns among treatment groups resembled differences in mortality (Fig. 2). Skin temperature data were successfully collected for 113 bats (77%). An analysis of variance (ANOVA) showed that average torpor bout duration varied with *Pd* inoculation (*F*_4, 102_ 23.0, *P*<0.001) and hibernation temperature (*F*_1, 102_  = 115, *P*<0.001). Bats exhibited longer torpor bouts at 4°C, and bats in the control groups had longer torpor bouts than bats in each inoculation treatment (*P*<0.05; Fig. 2). Additionally, bats inoculated with 500 conidia also exhibited shorter torpor bouts than bats inoculated with 500 000 conidia (*P*<0.05). There was a significant interaction between *Pd* inoculation and temperature (*F*_4, 102_  = 6.6, *P*<0.001) reflecting a larger decrease in average torpor bout duration associated with *Pd* inoculation at 10°C (Fig. 2). There was no significant effect of sex on torpor bout duration (*F*_1, 103_  = 3.5, *P* = 0.063), although the observed power was low (0.46), due to low sample sizes and high variance for each sex within groups. Comparison of control males and females (Fig. 3) revealed longer torpor bouts among females hibernating at 4°C (*t* = −1.984, df  = 12, *P* = 0.04) but not 10°C (*t* = −1.302, df  = 9, *P* = 0.11). Overall, we found that *Pd* inoculation, especially at low doses, resulted in an increase in arousals from hibernation.

**Figure 4:**
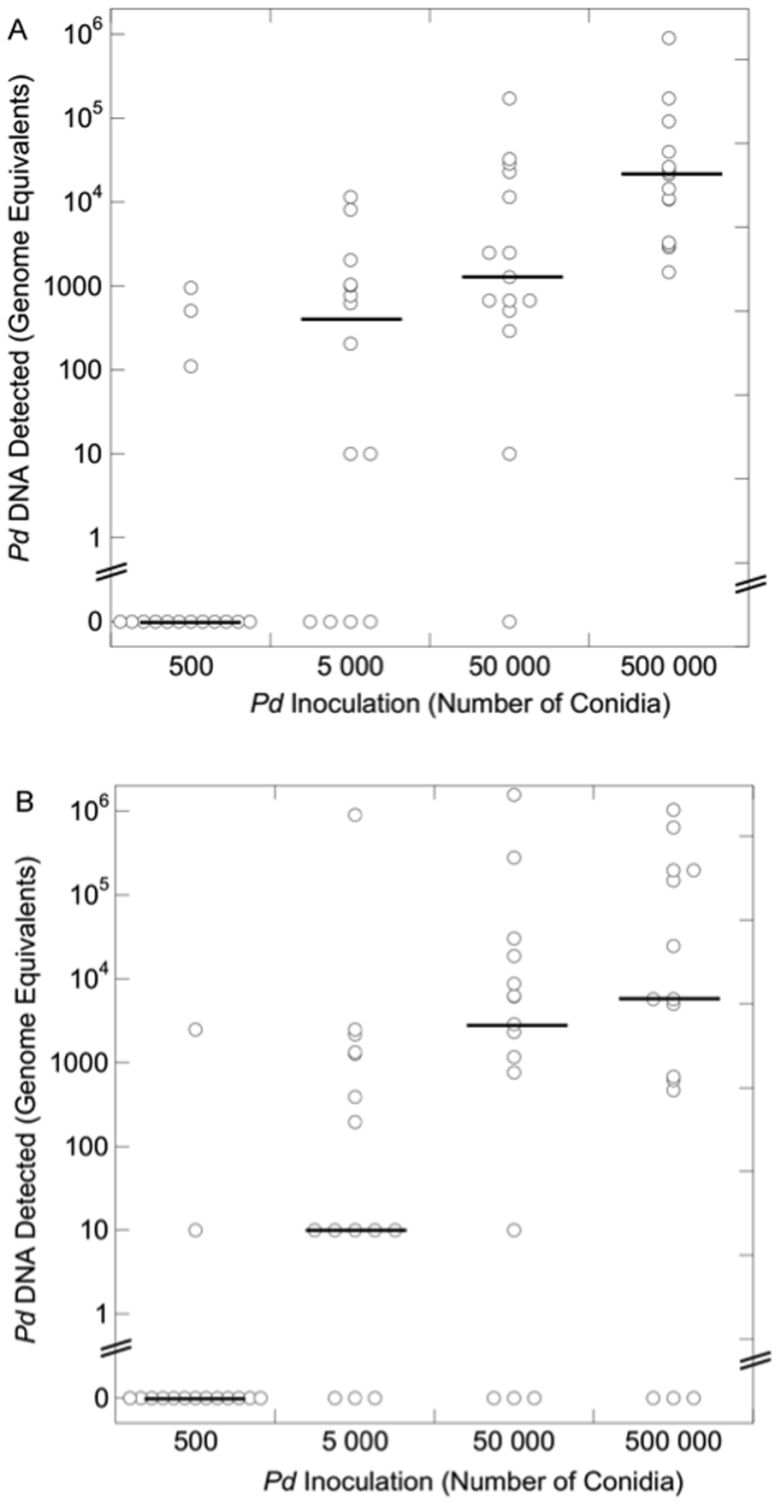
*Pseudogymnoascus destructans* (*Pd*) DNA detected at the end of hibernation on little brown myotis (*Myotis lucifugus*) inoculated with varying doses of *Pd* conidia and hibernated for five months at either 4°C (A) or 10°C (B). Individual observations are represented with open circles and medians represented by horizontal lines. At both temperatures, significantly less *Pd* was detected on bats in the 500 conidia group than on bats in other treatment groups. At 4°C, less *Pd* was detected on bats inoculated with 5 000 conidia compared to bats inoculated with 500 000.

Greater amounts of *Pd* detected on wing swabs were not associated with higher mortality or more frequent arousals from torpor. *Pd* was not detected by quantitative polymerase chain reaction (qPCR) on any bats (*n* = 147) upon arrival at our facility, and was not detected on any control bat at the end of hibernation (*n* = 29). *Pd* loads detected on inoculated bats at the end of hibernation were highly variable (Fig. 4). We found no difference in the median fungal loads detected on bats in each inoculation treatment between temperatures. Within each temperature, however, median fungal loads varied significantly among inoculation groups (4°C: *H* = 35.4, *P*<0.001; 10°C: *H* = 22.6, *P*<0.001; Fig. 4). At both temperatures, significantly less *Pd* was detected on bats inoculated with 500 conidia compared to all other treatment groups. At 4°C, less *Pd* was detected on bats inoculated with 5 000 conidia compared to bats inoculated with 500 000.

Inoculated bats surviving the hibernation experiment showed marked declines in *Pd* loads within several weeks of removal from hibernation. Forty-five inoculated bats were swabbed both upon removal from hibernation as well as 19 days later. *Pd* was detected on neither date for 8 bats (18%), and *Pd* loads declined to zero in 16 bats (35%). The remaining 21 bats (47%) exhibited a 15-fold median decrease in *Pd* load, with a median load of 11 487 genomic equivalents (range  = 292–640 000) at the end of hibernation, followed by a median load of 735 genomic equivalents (range: 10–18 611) 19 days later.

## Discussion

We found that WNS mortality is influenced by the level of *Pd* exposure, characteristics of the host, and the environment, and that several variables have interacting effects. As we predicted, differences in torpor patterns mirrored differences in mortality, but contrary to our expectations, bats inoculated with the lowest *Pd* dose experienced the greatest mortality rate and shortest torpor bouts during our study. Together, these data provide important insights on WNS survivors and have several implications for the possibility of long-term survival of little brown myotis in eastern North America.

Bats with greater body condition, indicative of greater fat reserves [34], were more likely to survive our experiment. Because bats rely on the metabolism of fat to arouse from torpor and sustain brief periods of euthermy during hibernation [4], fat reserves limit the number of times a bat can arouse [35]. Thus, bats with greater body condition at the onset of hibernation can sustain more arousals during the course of a winter, making them better suited to surviving the increased frequency of arousals associated with WNS [13], [17]. In a study of free-ranging bats affected by WNS, however, Reeder and colleagues [17] found no relationship between date of death and body condition. This discrepancy likely results from the confounding effects of other pertinent variables influencing the disease. As our results show, WNS mortality is driven by the interaction of variables pertaining to not only the host, but also the pathogen and the environment. Understanding how our results, obtained under carefully controlled conditions, compare to survival and mortality of wild populations requires the incorporation of all these variables, and provides a foundation for hypotheses related to the persistence of little brown myotis in the WNS-affected region of North America.

Females also exhibited greater survival probability in our study. Female little brown myotis are frequently documented with greater mass or body condition compared to males in the late fall or early winter [17], [27], [36], [37], a difference also present in our captive sample. Although females in our study had greater body condition than males at the onset of hibernation, we did not find a large correlation between sex and body condition in our survival analysis, demonstrating that while large body condition contributes to survival in females, there are other sex-based differences contributing to variation in mortality. Jonasson and Willis found that hibernating little brown myotis females have less pronounced declines in body mass over winter compared to males [26], [27], but were unable to attribute this to differences in torpor patterns during hibernation between the two sexes. We were also unable to detect differences in mean torpor bout duration between males and females in our overall analysis, although statistical power was low. A limited comparison of torpor bouts between males and females in control groups did reveal differences in torpor behaviors, however, potentially explaining why females had higher survival rates than males. This was true of inoculated as well as control bats; 83% (*n* = 5) of the mortality observed among control bats hibernated at 10°C consisted of males with body condition below that of any female in the group.

Differences in winter body condition and torpor behaviors between male and female little brown myotis are believed to be related to the reproductive biology of the species. Because copulation occurs throughout fall and winter, and ovulation occurs in spring after emergence from hibernation, several have argued that males benefit from a winter torpor strategy favoring frequent arousals while females arouse less frequently to emerge from hibernation with the fat stores necessary for ovulation [27], [38]–[42]. While some data support this hypothesis [27], the large variation observed in winter torpor behavior provides evidence that each sex exhibits diversity in their torpor behaviors [27]. Furthermore, arousal from hibernation and energy savings while torpid are not only determined by sex. Frequency of arousals and torpid metabolic rates decrease with temperature, resulting in greater energy savings [30], [31]. Boyles and colleagues suggested that both sexes of little brown myotis select microclimates within caves for hibernation based upon their body condition, i.e. bats with less fat hibernating in colder regions to conserve energy [25]. Thus, torpor patterns in free-ranging little brown myotis are influenced by the interaction of numerous variables, including sex, body condition, and environmental conditions.

Because colder temperatures are conducive to greater energy savings for bats (provided ambient temperature remains above the hypothalamic set-point) [4], [31] and are associated with slower fungal growth [8], we predicted that WNS mortality would be greater at higher temperatures. This was supported by our mortality and torpor duration results, the latter of which found a significant interaction between *Pd* inoculation and temperature, and is consistent with population declines observed in little brown myotis hibernacula, where warmer hibernacula exhibited the largest declines [28]. Similarly, we hypothesized fungal loads would be greater at 10°C, but contrary to our expectations, we did not detect differences in *Pd* loads between temperatures. Thus, *Pd* loads appear to be poor indicators of the severity of infection and WNS, as both mortality and frequency of arousals from hibernation increased at 10°C. It is important to note, however, that because 90% relative humidity was maintained in environmental chambers at both temperatures, the absolute humidity of the air was approximately 40% greater at 10°C. This difference in absolute humidity between temperatures could potentially result in different progressions of WNS, resulting in differences in the rates of evaporative water loss in bats or fungal invasion of the skin [43]. Thus, the role of absolute humidity was unclear from our experiment. Regardless, the high variability in *Pd* loads detected at both temperatures highlights the variability in *Pd* growth on bats relative to growth patterns in culture. In free-ranging bats exposed to more variable initial *Pd* exposures than those used in our experiment, and inhabiting hibernacula with conditions that can fluctuate throughout the winter, change in fungal loads are likely to be even more variable. In addition to being more variable, exposure to *Pd* in free-ranging bats is likely to occur repeatedly during the winter, as bats move about within and among hibernacula. These dynamics of *Pd* spread are poorly understood, however, and more research in this area is needed.

Also contrary to our prediction, we observed the greatest mortality and shortest torpor bouts in bats inoculated with the least concentrated solution of *Pd* conidia. This paradoxical result could be explained if lower concentrations of *Pd* grow differently than *Pd* at high densities. We hypothesize that *Pd* germination is inversely related to the density of conidia, resulting in more rapid fungal invasion and mortality in bats inoculated with 500 conidia. Density-dependent growth has been documented in many fungal species and is known as autoinhibition or self-inhibition, a process that can be mediated by volatile organic compounds produced by multiple genera of fungi [44], [45]. In the first study to show inoculation of bats with *Pd* causes WNS, Lorch and colleagues [3], [13] noted that their captive study was not long enough to result in mortality despite histological evidence of infection among inoculated bats. Density-dependent growth of *Pd* may explain why mortality did not occur within the time period of their study, which inoculated bats with 500 000 conidia. Our mortality data are only suggestive of self-inhibition in *Pd*, however, and research documenting germination at varying concentrations of conidia is needed to directly address this hypothesis. Such research, along with studies documenting natural exposure dynamics among free-ranging bats, are needed to better inform captive studies of WNS, which typically inoculate bats with 500 000 conidia [3], [13]. Inoculations resulting in mortality patterns that differ from wild populations may produce misleading insights into WNS.

It is notable that we were often unable to detect *Pd* DNA on swabs from bats inoculated with 500 conidia. This demonstrates that the number of *Pd* conidia did not exponentially increase on bats in this treatment. We hypothesize that bats have some ability to control the fungal infection at this level of exposure. Although the mechanism of control is uncertain, the increased frequency of periodic arousals observed in these treatments likely plays some role. Arousals provide opportunities for euthermic rest, grooming [46], and immune upregulation, although the brevity of periodic arousals in bats compared to other hibernating mammals likely limits potential immune responses to *Pd* [47]–[49]. As previously discussed, however, the number of arousals bats can energetically sustain are limited, and the frequent arousals in bats inoculated with 500 conidia resulted in high mortality despite an ability to control the fungus. Furthermore, *Pd* always remained on some bats within the 500 conidia treatment groups, serving as vectors for continued *Pd* exposure within this hibernation chamber.

Mortality in the remaining inoculation treatments was not significantly greater than controls in our model. This lack of difference was driven by the low mortality observed in the remaining inoculation treatments hibernated at 4°C (33–47%) and relatively high mortality in the 10°C control group (43%). The mortalities in the 10°C control group (*n* = 6) are well explained by the logistic regression model. Mortalities in this group were primarily (83%; *n* = 5) males with body condition indices at the onset of hibernation that were below the median body condition. It is well documented that lower temperatures are more energetically favorable for hibernating bats [4], [31], a conclusion supported by our own data. Thus, it is not surprising that we observed high mortality among male bats, which aroused more frequently from hibernation, with low fat reserves when placed in an energetically unfavorable environment. Mortality among bats with low body condition in both control groups may also result from placing bats in environmental conditions that differ from their native hibernacula. The 4° and 10°C environmental chambers represented temperatures that are colder and warmer, respectively, than both of the hibernacula we sampled in Illinois and Michigan. Research with captive big brown bats found that although hibernating bats conform to temperatures inside of environmental chambers, torpid metabolic rates are influenced by the temperature regime bats were accustomed to in their native hibernacula [50]. As a result, conditions inside both chambers may be more energetically stressful than can be predicted based upon temperature alone. Regardless of why some mortality occurred in the control groups, the lack of difference in mortality between the control and treatment groups exposed to >500 conidia should not be interpreted to mean that bats in these treatments did not have WNS. To the contrary, bats in all inoculation treatment groups at both temperatures exhibited significantly shorter torpor bouts than controls, a key sign of WNS [13], [17]. The reduction in torpor bout length demonstrates that bats in all inoculation treatments developed one of the hallmarks of WNS and would exhaust their energy reserves in a longer hibernation period, unlike bats in the control group that had normal torpor bout lengths, but our results show this mortality would occur after bats exposed to a smaller number of conidia early during hibernation. It is important to note that we did not use histopathologic criteria to confirm WNS in bats in our experiment, and assumed that *Pd* inoculation was the cause of the increased frequency of arousals and increased mortality compared to control bats, an assumption that is strongly supported by recent research [3], [13].

At the northern edge of their range, little brown myotis are reported to hibernate for two months longer than the duration of our experiment [37]. Thus, the ability of free-ranging bats to survive exposure to *Pd* must be considered in the context of winter duration and hibernaculum temperature. Our model predicts that little brown myotis with greater body condition indices inhabiting the regions of North America where the hibernation period lasts approximately 5 months will be able to persist in *Pd*-contaminated hibernacula, provided bats have access to cold roosting microclimates. Although the maximum winter duration little brown myotis can survive with *Pd* is uncertain, hibernacula temperatures below those included our study may confer even greater survival benefits.

Variables relating to the environment, host, and pathogen interact to produce disease [32]. Our study presents WNS survival and mortality within the context of the disease triangle, showing that little brown myotis females, and individuals of both sexes with higher body condition, are more resilient to *Pd*, and that cold hibernacula further increase individual odds of survival. These results suggest a scenario in which little brown myotis may continue to persist in the affected region of North America, with selection favoring individuals with large fat reserves and preference for cold hibernation sites. Because our study was conducted with naïve individuals under controlled conditions, however, additional research on survival in free-ranging populations, and the possible role of the immune system in pathology or resistance, are needed to better understand the fate of little brown myotis and other cave-hibernating species in eastern North America.

## Materials and Methods

### Animal Collection

This study was carried out on non-endangered animals in strict accordance with the recommendations in the Guide for the Care and Use of Laboratory Animals of the National Institutes of Health. All methods were approved by the Institutional Animal Care and Use Committee at Bucknell University (protocol number DMR-016). Animals were collected at Blackball Mine in Utica, Illinois, USA by state wildlife officials (including JAK with Illinois Department of Natural Resources) on non-endangered bats; thus numbered permits were not required or issued. Animals were collected at Iron Mountain Iron Mine in Vulcan, Michigan, USA under Scientific Collector' Permit SC 1475 from the Michigan Department of Natural Resources to DMR. In accordance with the permit and with state wildlife policies, research was either conducted on state land or on private property, with the explicit permission of private landowners.

We collected 147 hibernating little brown myotis (70 male; 77 female) from Blackball Mine and Iron Mountain Mine on 2–3 November, 2013. Bats were placed in individual cloth bags and transported to Bucknell University in Pennsylvania inside a portable refrigeration unit (Dometic Ltd., Bedfordview, South Africa) set to an internal temperature of 4°C. We determined the sex, weight, and right forearm length of each bat upon arrival at the laboratory and determined the body condition of each bat by dividing the mass by the forearm length [34]. Although the hibernacula that bats were collected from were believed to be unexposed to *Pd*, we swabbed the wings and muzzle of each bat with a sterile cotton swab to collect any *Pd* cells in order to verify that bats had not been exposed. Both wings were swabbed five times on both the dorsal and ventral sides. All bats were fitted with modified iButton temperature dataloggers (Embedded Data Systems, Lawrenceburg, KY, USA), programmed to record skin temperature (*T*_sk_) at 30-min intervals [17].

### Fungal Inoculation and Hibernation

Bats were placed into treatment groups representing the number of *Pd* conidia that bats were to be inoculated with prior to being placed into hibernation: 0 (control), 500, 5 000, 50 000, or 500 000 conidia. The *Pd* culture was derived from an isolate from an infected little brown myotis in Pennsylvania in 2010. Conidia were enumerated using a hemocytometer and 0.25% Trypan Blue staining, and viability of spores was confirmed by culture on Sabouraud agar plates. Each group was replicated once at 4 and once at 10°C. To the extent possible, we randomly selected an equal number of males and females from each hibernaculum to be placed into each treatment group (*n* = 14–15 bats per treatment). Once separated into treatment groups, each bat was either sham inoculated (controls) with 50 µL phosphate buffered saline with 0.05% Tween-20 (PBST) or inoculated with the appropriate number of *Pd* conidia suspended in 50 µL PBST. The solution was pipetted onto the ventral surface of one wing below the wrist, and distributed along the wing by gentle manipulation of the wing. Similar captive inoculation methods have been clearly demonstrated to cause WNS in recent studies [3], [13]. Bats in each treatment group were housed together in open air aluminum cages (Zoo Med Laboratories Inc., San Luis Obispo, CA, USA), provided with *ad lib* water, and placed into environmental chambers set to maintain a constant temperature (4 or 10°C) and ≥90% relative humidity. Control and inoculated treatment groups were housed in separate environmental chambers. Within the chamber housing inoculated treatment cages, individual cages were not in contact with one another, preventing any contact among bats, and, therefore, *Pd* transmission among cages. A study with a similar design found that *Pd* transmission did not occur between cages of inoculated and un-inoculated bats when cages were separated within the same environmental chamber [3]. Temperature and relative humidity dataloggers (TransiTempII, MadgeTech, Warner, New Hampshire, USA) were placed inside each chamber to confirm environmental conditions. To avoid disturbance and unnatural arousals from hibernation, chambers were only opened once per month to provide fresh water and remove any moribund bats.

All bats were swabbed a second time following removal from hibernation to estimate *Pd* loads. Bats were left in hibernation for ca. 5 months (148 d), after which dataloggers were removed from all bats, and surviving individuals were placed in an indoor flight cage where they were hand-fed gut-loaded mealworms until able to self-feed. Conditions inside the flight cage were maintained at approximately 21°C and 60% relative humidity. Inoculated bats surviving hibernation were swabbed a third time 19 d after the end of hibernation to determine the change in *Pd* loads after bats were removed from an environment favorable for the growth of the fungus. Because surviving bats were not euthanized in this experiment, and moribund bats were only removed once per month, typically several days or weeks after mortality, no tissues were available for a histological confirmation of WNS [2].

### Quantifying Fungal DNA

We used qPCR to determine *Pd* loads on bats prior to and following emergence from hibernation. To prepare for genomic DNA (gDNA) extraction, swabs were incubated at 37°C for 30 minutes in Tris-EDTA buffer (10 mM Tris, 1 mM EDTA; Amresco, Solon, Ohio, USA) containing 20 U/ml Lyticase (Sigma-Aldrich, St. Louis, Missouri, USA) and 30 mM Dithiothreitol (Sigma-Aldrich). Genomic DNA was extracted using the QIAamp DNA Micro kit (Qiagen Inc., Valencia, California, USA), following the manufacturer's instructions, including the addition of 1 µg carrier RNA.

We used a Taqman 5′ endonuclease assay targeting the IGS region of the rRNA complex to detect *Pd* gDNA extracted from swabs. Primers were synthesized by Integrated DNA Technologies (Coralville, IA, USA) using the sequences [51]: forward primer nu-IGS-0169-5'Gd: 5'– TGC CTC TCC GCC ATT AGT G –3'; reverse primer nu-IGS-0235-3'–Gd: 5'– ACC ACC GGC TCG CTA GGT A –3'; and probe nu-IGS-0182/0204-Gd: 5'– (FAM) CGT TAC AGC TTG CTC GGG CTG CC (BHQ-1) –3'. Each 25 µL PCR reaction contained 12.5 µL Bio-Rad 2× Supermix (Hercules, California, USA), 10.5 µL sample elution, 1 µL of each primer (0.4 µM), and 1 µL probe (0.2 µM). Reactions were performed using a Bio-Rad iCycler starting with an initial 3 min incubation at 95°C, followed by 40 cycles of 30 s at 95°C and 30 s at 60°C. As a control, unused swabs known to be negative for *Pd*, and swabs exposed to a known quantity of *Pd*, were included on each plate, as well as no-template control. In order to quantify gDNA on swabs, we created standards by spiking swabs with 10 or 10 000 conidia. Standards were purified in parallel with each batch of samples and run in triplicate on each PCR plate.

The cycle threshold (Ct) was determined using the thresholds set by the data analysis software (iCycler iQ version 3.0a). The average Ct for a swab spiked with 10 *Pd* cells was 34.8 (*n* = 34), and the assay was found to be linear at all Ct values lower than this. A Ct of 38.1 was calculated to represent 1 conidia and was used as the limit of detection for the assay. All swabs with non-exponential fluorescence increases or with a Ct between 34.8 and 38.1 Ct were considered ambiguous and reanalyzed to confirm that the amount of *Pd* detected was greater than 1 conidia. Swabs with ambiguous results in two analyses were not considered (*n* = 3). All samples with final Ct values between 34.8 and 38.1 were considered to have a *Pd* load of ≤10 genomic equivalents, while samples with Ct values greater than 38.1 were considered *Pd*-negative. The amplification efficiency of the PCR reaction was calculated to be ∼100% based on the slope of the standard curve. Swabs with a Ct less than 34.8 were used to calculate the number of conidia present according to the formula: 10 000×2^Ct(exp)-Ct(ss)^, where *exp* is a swab sample from a bat and *ss* is a swab spiked with 10 000 conidia.

### Data Analysis

Mortality and survival were analyzed using a binary (logit function) logistic regression model including temperature (categorical), *Pd* inoculation (categorical), sex (categorical), and body condition (scale) as dependent variables. To aid interpretation of results, body condition values (range: 0.161–0.269) were multiplied by 100 prior to inclusion in the model. We did not include the state of origin (Michigan and Illinois) as a variable because within each sex, preliminary analyses found that the body condition did not differ between states (two-tailed t-tests, *P*>0.05) and because mortality rates were similar (Illinois: 46%; Michigan: 48%). *T*_sk_ data recorded at 30-min intervals were analyzed to characterize the torpor behavior of bats during hibernation. For each bat, we determined the date and time for each arousal from torpor and calculated the average duration of torpor bouts. Bats were considered aroused from torpor when *T*_sk_ was ≥20°C for ≥1 reading, or when *T*_sk_ ≥15°C for ≥2 readings. We compared the average duration of torpor bouts with an ANOVA using temperature, inoculation, sex, and a temperature-inoculation interaction as main effects to test our hypotheses regarding torpor behaviors. Due to low statistical power for the variable sex, we also conducted two 1-tailed *t*-tests comparing the duration of torpor bouts of males to females at each temperature. Data for torpor bout duration were transformed by calculating the natural logarithm prior to analysis to meet statistical assumptions. *Pd* loading data could not be transformed to meet assumptions of normality and were compared among inoculation treatments using separate Kruskal-Wallis tests for each hibernation temperature. Means comparisons were made using a Wilcoxon test for each pair of treatments, with a sequential Bonferroni-Holm correction [52]. *Pd* loads were compared between temperatures using a Wilcoxon test for each inoculation treatment. Control groups were not included in *Pd* load analyses, because *Pd* was never detected on control animals. All tests used a significance threshold of 0.05 and Fisher' Least Significant Difference to compare means where appropriate.
